# Autonomic Painful Diabetic Gastropathy Presenting As Recurrent Episodic Abdominal Pain in a Young Man With Type 1 Diabetes: A Case Report

**DOI:** 10.7759/cureus.106408

**Published:** 2026-04-03

**Authors:** Simha Swaraj Sirivela, Nifson Yusuf, Numan A Mandaty, Kiran G Kulirankal, Merlin Moni

**Affiliations:** 1 College of Medicine, Amrita Institute of Medical Sciences, Kochi, IND; 2 Internal Medicine, Amrita Institute of Medical Sciences, Kochi, IND

**Keywords:** abdominal pain, autonomic dysfunction, clonidine, diabetic autonomic neuropathy, diabetic gastropathy, endocrine disorders, painful gastropathy, tilt table testing, type 1 diabetes mellitus (t1d)

## Abstract

Autonomic painful diabetic gastropathy is an uncommon and often overlooked cause of recurrent abdominal pain in individuals with diabetes. We describe a young man with type 1 diabetes who developed repeated episodes of severe epigastric pain and vomiting after a traumatic event, with all routine investigations failing to identify a structural, metabolic, or functional cause. His symptoms were resistant to standard therapies but showed rapid and sustained improvement with centrally acting sympatholytic treatment, and autonomic testing later supported underlying dysautonomia. This case highlights the importance of considering autonomic gastrointestinal involvement in unexplained, recurrent abdominal pain in diabetic patients, as early recognition can guide targeted therapy and help avoid unnecessary investigations and hospitalizations.

## Introduction

Diabetic autonomic neuropathy of the gastrointestinal tract represents a complex spectrum of motility and sensory disturbances resulting from chronic injury to the autonomic fibers regulating gastrointestinal function. Early descriptions highlighted that gastrointestinal involvement may manifest not only as delayed gastric emptying but also visceral hypersensitivity, dysregulated gastric accommodation, and pain-predominant presentations that may occur even with normal gastric emptying studies [1,2]. Gastroparesis and related autonomic gastrointestinal disorders are increasingly recognized to present with episodic or cyclical symptoms, including abdominal pain, nausea, and vomiting, particularly in patients with type 1 diabetes [3,4].

The pathophysiology involves impaired vagal modulation, sympathetic overactivity, and altered afferent signaling, leading to abnormal perception of gastrointestinal stimuli and fluctuating symptom severity over weeks to months [2-4]. Pain-predominant autonomic gastropathy, although less common than classical gastroparesis, is characterized by severe epigastric pain that may mimic intra-abdominal emergencies, often accompanied by vomiting and autonomic instability despite unremarkable laboratory and imaging studies [1,5]. As a result, diagnosis is frequently delayed.

In the present case, a young man with type 1 diabetes developed recurrent monthly episodes of severe abdominal pain and vomiting shortly after a road traffic accident (RTA). While direct literature linking trauma to painful diabetic gastropathy is limited, acute physiologic stress is known to unmask latent autonomic dysfunction by triggering sympathetic surges and destabilizing autonomic homeostasis [2,6,7]. This case underscores the need to consider autonomic painful diabetic gastropathy in young diabetic patients with recurrent severe abdominal pain when structural, metabolic, and inflammatory etiologies have been excluded. Recent cohort data further highlight the substantial symptom burden, fluctuating gastrointestinal patterns, and episodic nature of autonomic gastrointestinal dysfunction in diabetes, underscoring its rising clinical relevance [8].

## Case presentation

A 24-year-old man with type 1 diabetes mellitus, diagnosed at the age of 16 years, and with a history of RTA with polytrauma six months prior, presented with a six-month history of severe, episodic epigastric pain accompanied by recurrent vomiting. These attacks occurred approximately once every month, each severe enough to require hospital admission. The pain was acute in onset, intense, non-radiating, and associated with tachycardia, hypertension, diaphoresis, and agitation, while the patient remained completely asymptomatic between episodes.

Laboratory evaluation across multiple admissions consistently demonstrated normal complete blood counts, liver and renal function tests, electrolytes, inflammatory markers, and pancreatic enzymes. Glycemic assessment indicated suboptimal control, with an HbA1c of 9%. Serum ketones were trace to negative, with no biochemical evidence of diabetic ketoacidosis. Thyroid profile, morning cortisol, and urine porphyrins were within normal limits. Autoimmune screening, including antinuclear antibody by immunofluorescence assay (ANA-IFA), was negative. Toxicology workup, including serum lead levels and peripheral smear, showed no heavy-metal exposure. The patient had no history of drug abuse. A psychiatric assessment ruled out psychogenic or somatoform causes of pain.

Despite aggressive multimodal therapy including proton pump inhibitors (PPIs), antispasmodics, antiemetics, benzodiazepines, and high-dose nonsteroidal anti-inflammatory drugs (NSAIDs) and opioids, the patient experienced no meaningful relief and continued to have recurrent debilitating episodes.

**Figure 1:**
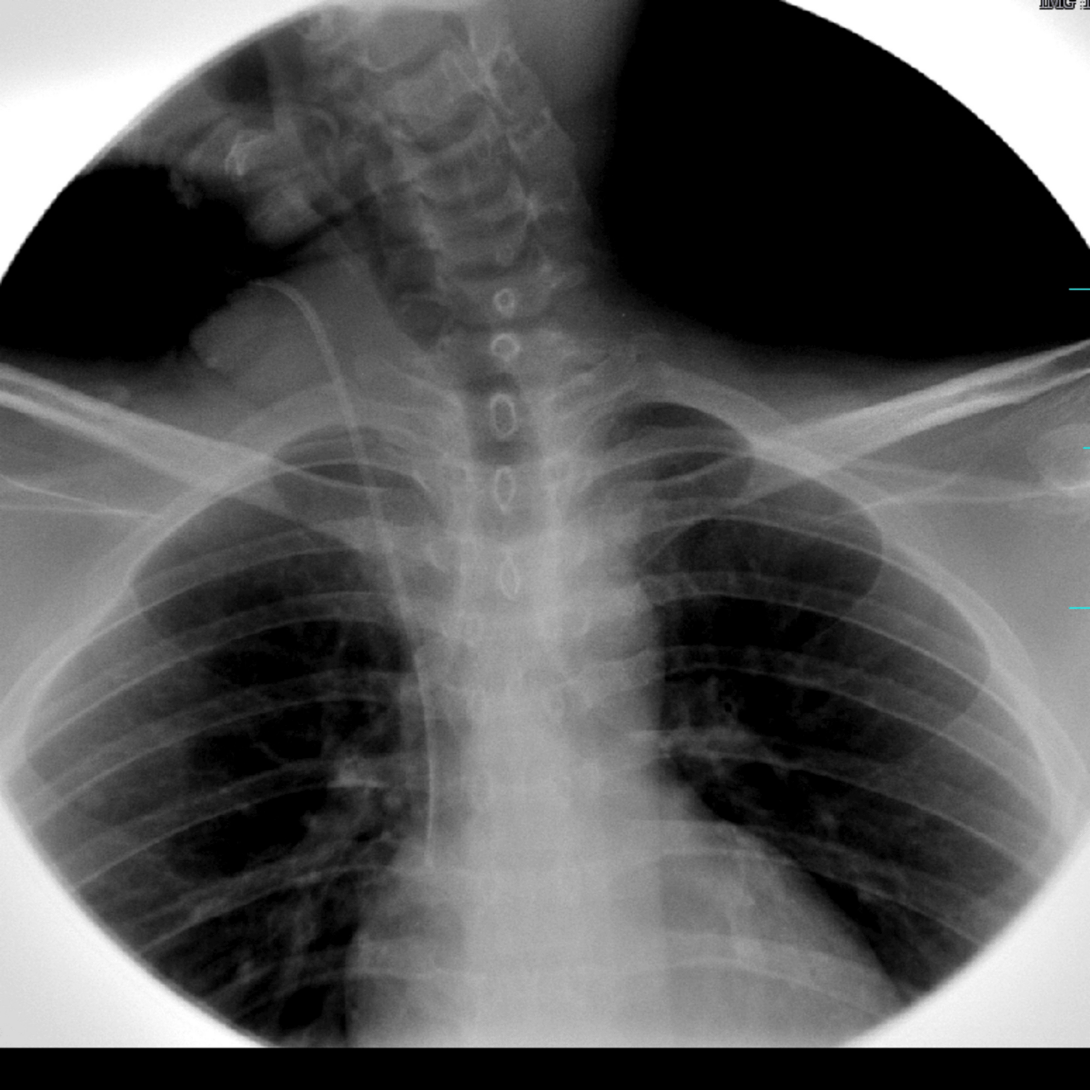
Contrast esophagogram showing normal passage of contrast with preserved esophageal contour and peristalsis, without evidence of stricture, obstruction, or gastroesophageal reflux.

**Figure 2:**
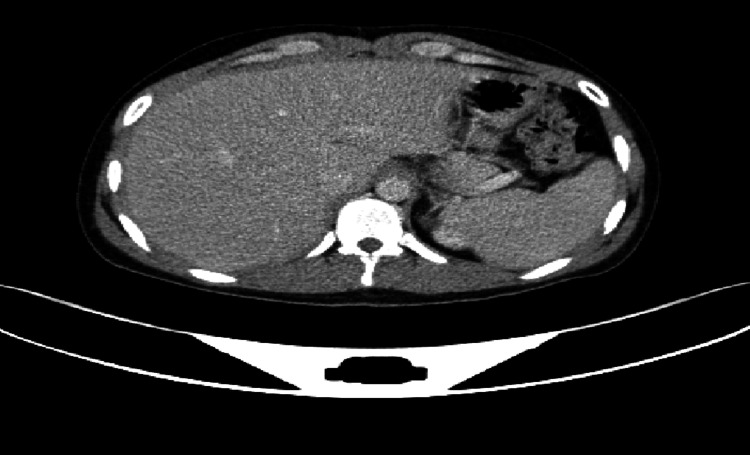
Contrast-enhanced CT of the abdomen demonstrating no significant abnormalities.

**Figure 3:**
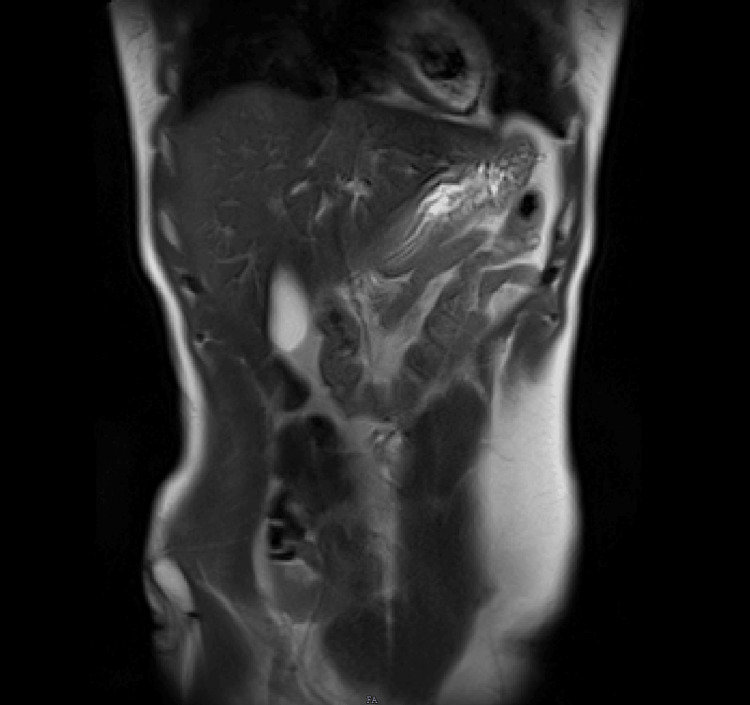
Coronal T2-weighted MRI sequences demonstrating normal signal intensity and morphology of the visualized structures, with no evidence of focal lesions or abnormal fluid collections.

**Figure 4:**
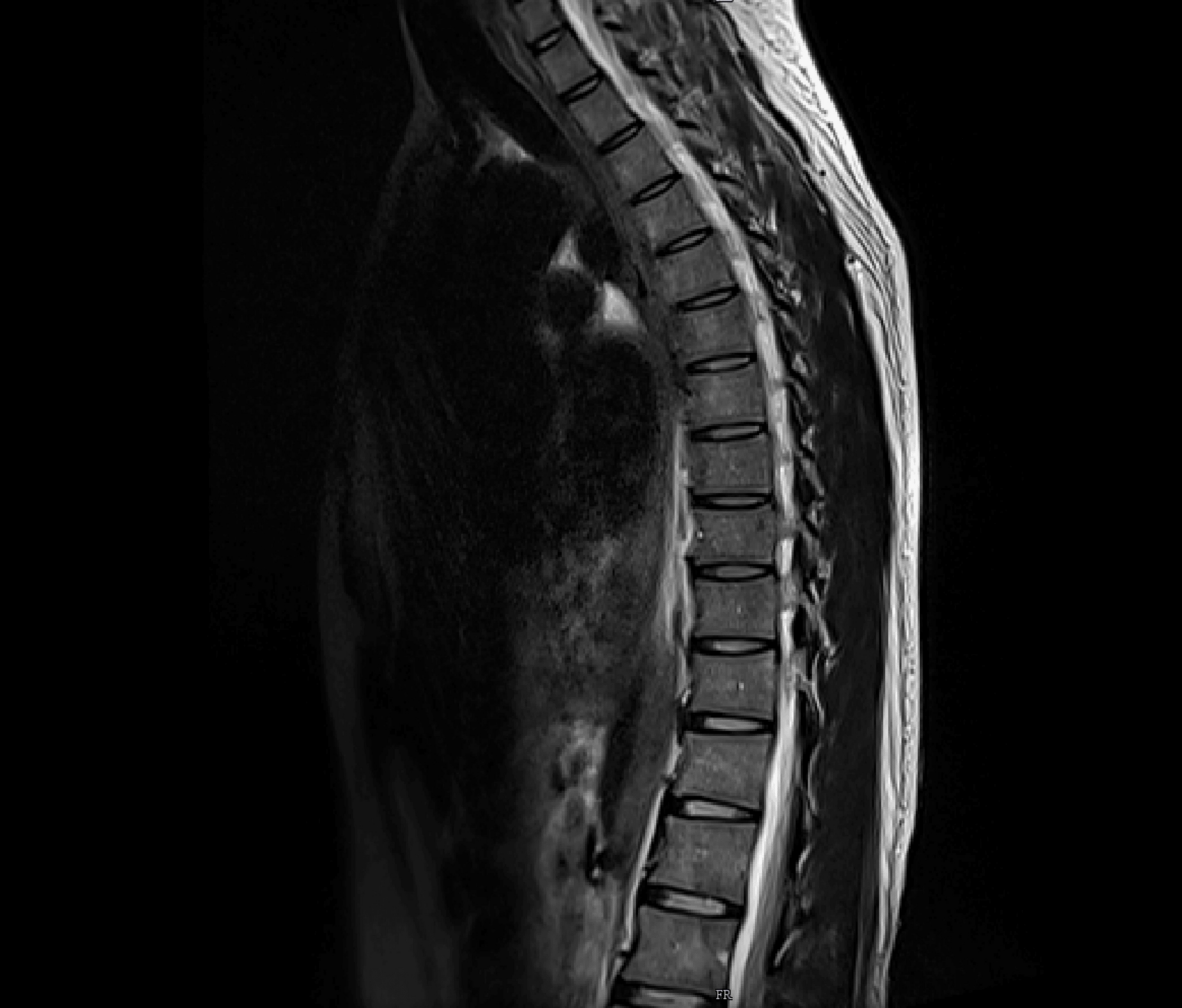
Sagittal MRI sequences of the thoracolumbar spine demonstrating no significant abnormalities.

Imaging studies were consistently unremarkable across all admissions. Upper gastrointestinal endoscopy and contrast esophagogram revealed normal mucosa with preserved peristalsis (Figure 1). Ultrasonography showed only mild hepatomegaly. Contrast-enhanced CT and MRI of the abdomen demonstrated no obstruction, inflammation, ischemia, or pancreatic pathology (Figures 2, 3). MRI of the thoracolumbar spine was normal (Figure 4).

**Figure 5:**
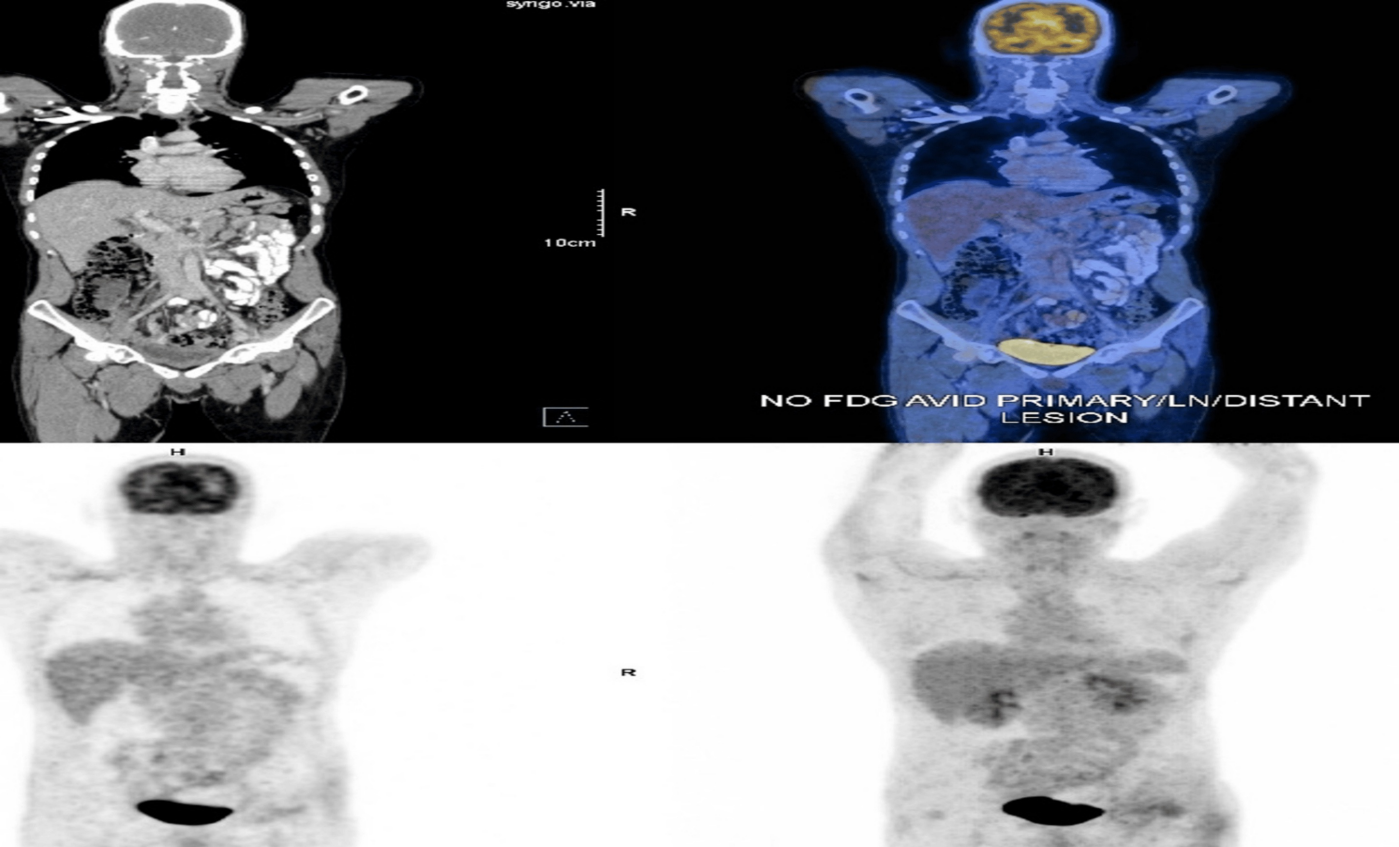
Whole-body FDG PET-CT demonstrating no metabolically active lesions. FDG PET-CT: fluorodeoxyglucose positron emission tomography-computed tomography

**Figure 6:**
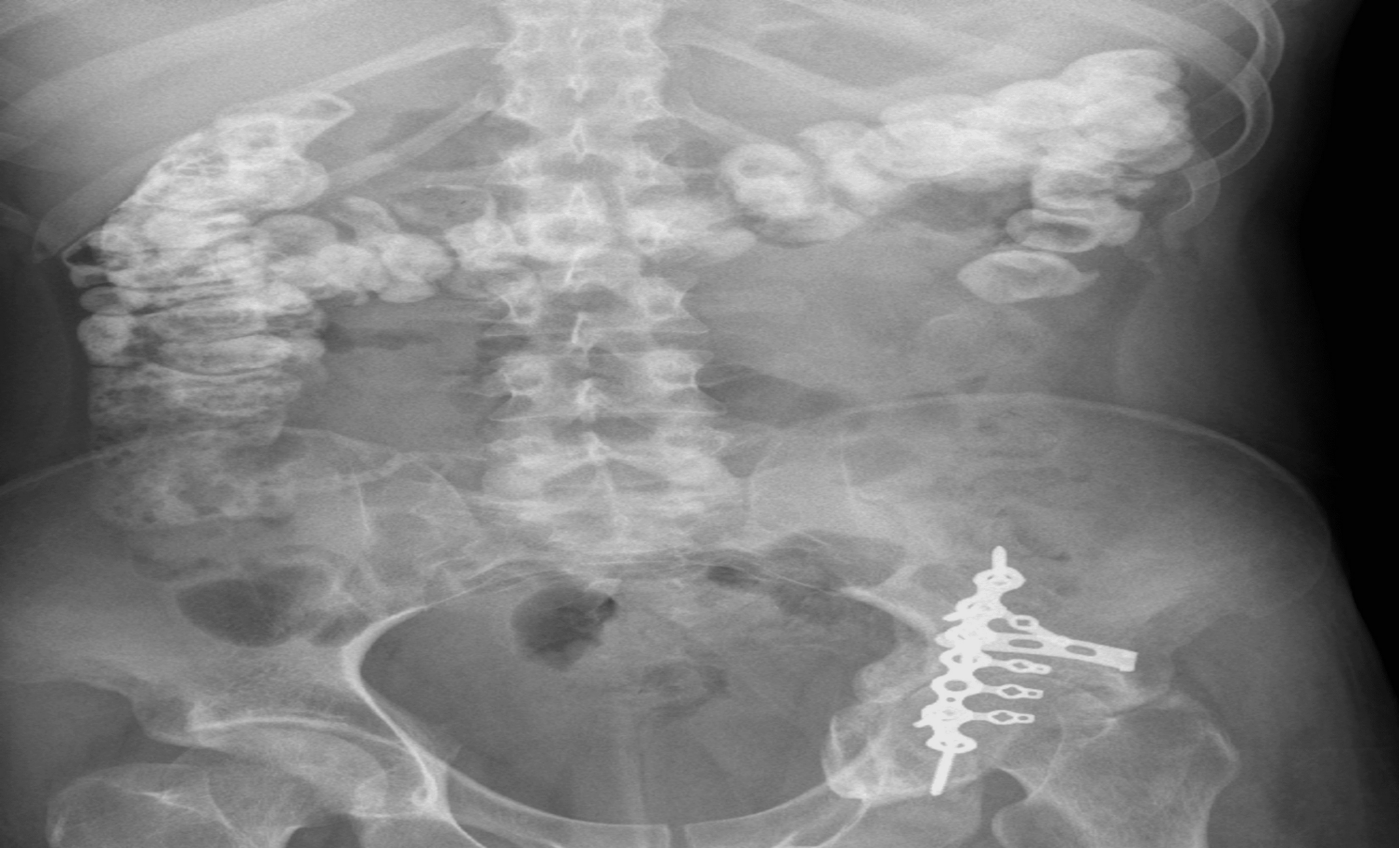
Oral Gastrografin study demonstrating normal transit of contrast through the bowel, with no evidence of obstruction, delayed passage, or leak.

During a subsequent admission, the patient again presented with severe epigastric pain, refractory vomiting, sinus tachycardia, and hypertension. A whole-body fluorodeoxyglucose positron emission tomography-computed tomography (FDG PET-CT) performed during this admission revealed no metabolically active primary, nodal, or distant lesions, and an oral Gastrografin study was also normal (Figures 5, 6). The patient’s pain failed to improve with conventional analgesics, and even escalation to opioid therapy, including morphine and fentanyl infusions, did not provide relief. A celiac plexus block performed during the same admission also failed to provide sustained benefit, arguing against isolated splanchnic nociceptive pathology. However, a trial of dexmedetomidine infusion resulted in rapid improvement in pain, vomiting, and autonomic instability, following which the infusion was gradually tapered and discontinued once symptoms stabilized; shortly thereafter, the patient again developed severe abdominal pain and vomiting.

Given the recurrent autonomic features despite normal structural evaluation, autonomic function testing was performed. A tilt table test demonstrated a drop in blood pressure from 114/63 mmHg to 84/39 mmHg and an increase in heart rate from 111 beats per minute (bpm) to 143 bpm when the patient was tilted to 70 degrees for 15 minutes, supporting autonomic dysfunction as a contributory mechanism. Based on these findings, the patient was started on oral clonidine 50 mcg three times daily, following which he experienced complete and sustained relief of abdominal pain, vomiting, tachycardia, and hypertension. On follow-up over a period of two months, he remained entirely asymptomatic with no recurrence of episodes.

## Discussion

Gastrointestinal autonomic neuropathy arises from progressive injury to autonomic fibers within the enteric nervous system and extrinsic sympathetic and parasympathetic pathways. Early foundational work demonstrated that patients with diabetic gastropathy often display impaired motility, abnormal visceral afferent processing, and dysregulated autonomic modulation, which may explain symptoms disproportionate to structural findings [1,2]. Later reviews reaffirmed that abnormalities in gastric accommodation, vagal impairment, and sensory hypersensitivity contribute significantly to pain-predominant phenotypes seen in clinical practice [3-5].

A broad differential diagnosis for recurrent episodic abdominal pain with vomiting in patients with diabetes includes diabetic ketoacidosis, acute intermittent porphyria, heavy metal toxicity such as lead poisoning, pancreatitis, mesenteric ischemia, bowel obstruction, and functional disorders, including cyclic vomiting syndrome and abdominal migraine. Importantly, diabetic autonomic neuropathy can present with pain-predominant gastrointestinal manifestations that mimic acute abdominal conditions despite the absence of structural pathology, making autonomic painful diabetic gastropathy an important diagnosis of exclusion in such clinical settings [5].

Cyclical symptom patterns, as seen in our patient, are well described in autonomic gastrointestinal disorders. Episodic exacerbations have been linked to glycemic fluctuations, autonomic instability, and stress-induced sympathetic surges [3,4]. These mechanisms explain why young diabetic patients may present with severe recurrent abdominal pain despite normal imaging, laboratory parameters, and endoscopic evaluations. The visceral hyperalgesia described in autonomic neuropathy further supports the occurrence of severe pain even in the absence of structural abnormalities [2,5].

The onset of symptoms following an RTA raises the possibility that acute trauma acted as a physiologic trigger or unmasked subclinical autonomic dysfunction. Sympathetic overdrive, catecholamine surges, and transient disruption of autonomic homeostasis following trauma are well documented and may precipitate dysmotility and visceral hypersensitivity in susceptible diabetic patients [2,6,7]. This mechanism aligns with the temporal sequence observed in our case.

The patient’s poor response to NSAIDs, opioids, and celiac block is consistent with neuropathic visceral pain, which is characteristically refractory to conventional analgesics [3,5]. The dramatic improvement with dexmedetomidine, a selective α2-adrenergic agonist, highlights the role of sympathetic overactivity in this condition. By reducing sympathetic tone, modulating visceral afferent transmission, and stabilizing autonomic output, dexmedetomidine produced complete symptom resolution, with sustained benefit on oral clonidine. This supports autonomic painful diabetic gastropathy as the unifying diagnosis.

Normal laboratory results, normal pancreatic enzymes, unremarkable CT and MRI imaging, a normal endoscopy, and negative porphyria evaluations ruled out metabolic, inflammatory, and surgical causes. The presence of orthostatic hypotension on autonomic testing strengthened the diagnosis of underlying autonomic dysfunction. Considering these findings, autonomic painful diabetic gastropathy should be incorporated early in the differential diagnosis when evaluating young diabetic patients with recurrent unexplained abdominal pain and vomiting.

Finally, recent data emphasize the substantial symptom burden and fluctuating gastrointestinal dysfunction experienced by patients with diabetic autonomic neuropathy, reinforcing the need for early recognition, appropriate autonomic testing, and targeted therapy [8].

## Conclusions

This case highlights the importance of recognizing autonomic painful diabetic gastropathy as a potential cause of severe, cyclical abdominal pain and vomiting in young patients with type 1 diabetes, particularly when routine laboratory studies, imaging, and endoscopic evaluations remain unrevealing. The temporal relationship to antecedent trauma suggests that acute physiologic stress may unmask previously silent autonomic dysfunction, precipitating recurrent debilitating symptoms. The patient’s poor response to NSAIDs and opioids, coupled with dramatic improvement following the use of central α2-agonists such as dexmedetomidine and clonidine, underscores the central role of sympathetic overactivity and visceral afferent dysregulation in this disorder. Early consideration of autonomic gastrointestinal neuropathy, along with targeted autonomic testing and individualized sympatholytic therapy, may prevent repeated hospitalizations and substantially improve quality of life. This case reinforces the need for heightened clinical suspicion, especially in young diabetics presenting with recurrent unexplained abdominal pain, to enable timely diagnosis and appropriate management.
